# Pelvic floor dysfunction and electrophysiology in postpartum women at 6–8 weeks

**DOI:** 10.3389/fphys.2023.1165583

**Published:** 2023-05-23

**Authors:** Jia-Cong Wu, Xiao-Li Yu, Hui-Jing Ji, Hai-Qin Lou, Hong-Ju Gao, Guo-Qin Huang, Xiao-Li Zhu

**Affiliations:** ^1^ Department of Obstetrics and Gynecology, Nantong Maternity and Child Health Care Hospital Affiliated to Nantong University, Nantong, China; ^2^ Department of Outpatient, Affiliated Hospital 2 of Nantong University, Nantong, China; ^3^ Nantong University Xinglin College, Nantong, China

**Keywords:** fatigue degree, muscle strength, pelvic floor dysfunction, pelvic floor muscle electromyography, pelvic organ prolapse, urinary incontinence, postpartum 6–8 weeks

## Abstract

**Objective:** To investigate the incidence of pelvic floor dysfunction (PFD) and electrophysiological indicators in postpartum women at 6–8 weeks and explore the influence of demographic characteristics and obstetric factors.

**Methods:** A survey questionnaire collected information about the conditions of women during their pregnancy and puerperal period and their demographic characteristics; pelvic organ prolapse quantitation (POP-Q) and pelvic floor muscle electrophysiology (EP) examination were conducted in postpartum women at 6–8 weeks.

**Results:** Vaginal delivery was a risk factor for anterior pelvic organ prolapse (POP) (OR 7.850, 95% confidence interval (CI) 5.804–10.617), posterior POP (OR 5.990, 95% CI 3.953–9.077), anterior and posterior stage II POP (OR 6.636, 95% CI 3.662–15.919), and postpartum urinary incontinence (UI) (OR 6.046, 95% CI 3.894–9.387); parity was a risk factor for anterior POP (OR 1.397,95% CI 0.889–2.198) and anterior and posterior stage II POP (OR 4.162, 95% CI 2.125–8.152); age was a risk factor for anterior POP (OR 1.056, 95% CI 1.007–1.108) and postpartum UI (OR 1.066, 95% CI 1.014–1.120); body mass index (BMI) was a risk factor for postpartum UI (OR 1.117, 95% CI 1.060–1.177); fetal birth weight was a risk factor for posterior POP (OR 1.465, 95% CI 1.041–2.062); and the frequency of pregnancy loss was a risk factor for apical POP (OR 1.853, 95% CI 1.060–3.237).

**Conclusion:** Pelvic floor muscle EP is a sensitive index of early pelvic floor injury. The changes in muscle strength and fatigue degree coexist in different types of postpartum PFD, and each has its own characteristics.

## Background

Pelvic floor muscles are the muscle groups at the bottom of the pelvis that connect the pubis and the coccyx. Decreased supporting ability of pelvic floor muscles and changes in the anatomical structure caused by various factors can result in pelvic floor dysfunction (PFD) (Grigoriadis et al., 2020). Pregnancy and delivery are closely related to the incidence of PFD (Groutz et al., 2004; Sievert et al., 2012). Hormone influence and excessive traction lead to the abnormality of the pelvic floor supporting structure, dominated by the levator ani muscle, which induces PFD. Most of these changes and lesions may be preventable or reversible if some risk factors for the development and progression of PFD can be controlled in the early postpartum period before the PFD symptoms appear, and PFD examination with treatment is executed on time (Bradley et al., 2007).

PFD is a heterogeneous disease with a complex pathophysiology and numerous clinical manifestations. Timely and finer evaluation of PFD is the key to effective treatment. At present, no assessment tool is considered the gold standard. The important evaluation methods are questionnaire surveys, gynecological visual examination, vaginal touch examination, electrophysiology (EP), and magnetic resonance imaging (MRI) (Pereira et al., 2014; Barbosa et al., 2018a; Frawley et al., 2021). Electrophysiological technology is used in electrical stimulation and biofeedback technology is used to collect biological electrical signals for diagnosis and treatment of diseases. EP is widely used in clinical practice because of its easy operation and rapid diagnosis. The strength and fatigue degrees of pelvic floor muscles are valuable basic pelvic floor electrophysiological indicators. The type, nature, location, and severity of potential PFD can be evaluated objectively and accurately, and the muscle strength of pelvic floor muscle groups can be quantified in the early stage through the detection of various indicators to provide a basis for rehabilitation treatment plans and evaluation of curative effects.

At present, there are few studies on the analysis of EP data on pelvic floor muscles based on a large sample of postpartum women (Xing et al., 2015; Yang et al., 2019), and the efficacy of the delivery mode on the pelvic floor function is also controversial (Ashton-Miller and Delancey, 2009; Slieker-ten Hove et al., 2009; Qian et al., 2016). In this study, the incidence of pelvic organ prolapse (POP) and urinary incontinence (UI) and the pelvic floor muscle EP in postpartum women at 6–8 weeks were investigated to explore their demographic characteristics and obstetric risk factors. Therefore, the results reflect accurately and quantitatively the pelvic floor function in women in the early postpartum period. The study was expected to provide a theoretical basis for the formulation of preventive measures for postpartum PFD.

## Participants and methods

### Participants

The study participants were women who underwent the first re-examination and voluntarily received pelvic floor muscle functional evaluation using EP 42 days after delivery in the outpatient clinic of the Maternal and Child Health Care Hospital Affiliated with Nantong University from June 2018 to February 2019. They were numbered according to the outpatient registration number, and their logs were also recorded. Eligible women were enrolled in the study group strictly according to the inclusion and exclusion criteria. This study was reviewed and approved by the medical ethics committee of the Maternal and Child Health Care Hospital Affiliated with Nantong University (No: Y2017004); participants voluntarily joined this study and signed the informed consent form.

Inclusion criteria: Postpartum women at 6–8 weeks with clean lochia, single birth, uncomplicated delivery, no history of chronic cough, asthma, long-term constipation or chronic pelvic pain, with no history of chronic diseases such as urinary tract infection, diabetes, hypertension, and kidney disease, and no history of pelvic surgery.

Exclusion criteria: Patients with multiple gestations, polyhydramnios or oligohydramnios, birth at the gestational weeks <28 weeks or ≥42, forceps delivery, a history of POP or UI before this pregnancy, and incomplete data.

## Methods

1. All participants completed a questionnaire based on general information, conditions during pregnancy and puerperal period, and had a routine gynecological examination. The participants were enrolled based on the inclusion and exclusion criteria.
2. Pelvic organ prolapse quantitation (POP-Q) staging and EP examination (Shanshan PHENIX U8, France) of pelvic floor muscles were carried out among the enrolled participants, including the collection of UI history, urinary incontinence questionnaire (ICI-Q-SF), gynecological examination, stress testing, Marshall–Bonney testing, and examination and staging of POP, involving strength and fatigue degree of pelvic floor muscles and vaginal dynamic pressure. A record of pelvic floor health was established. The clinical physical examination was completed by three obstetricians or gynecologists with a professional title of attending physician or above in the postpartum rehabilitation clinic, and the pelvic floor muscle EP examination was completed by two rehabilitation technicians who had been working in the postpartum rehabilitation clinic for more than 5 years.

### Specific steps for EP examination

The pelvic floor EP testing utilized an inflatable pressure assessment. The subjects were instructed to contract and relax their pelvic floor muscles. The pressure sensor converted their muscle movements into curves, allowing us to obtain EP indicators such as pelvic floor muscle strength, fatigue degree, and dynamic vaginal pressure.

The electronic pressure gauge was connected to the E port of the PHENIX host, and the line for monitoring the strength of abdominal muscle contraction was connected to the A port. The head of the treatment bed was raised by about 30–40°, and the patient rested on a disposable mat. Under the bladder lithotomy position, the vaginal probe balloon was gently inserted into the patient’s vagina. A syringe was used to inject 20–30 mL of gas slowly into the balloon without pain. Then the patient took a semi-sitting position (able to see the display screen) and was attached to the neutral electrode (three pieces in total, one placed at the iliac crest, and the other two placed on the surface of the abdominal muscles), avoiding scar tissue.

All patients were instructed to complete the following actions:

Perform three maximum-strength vaginal and anal contractions without using abdominal and leg strength, and the maximum dynamic pressure value of the pelvic floor muscles was obtained.

Perform three maximum-strength contractions of their abdominal muscles, and the strength of the muscle contractions in the abdomen was determined.

Rapidly contract and relax the pelvic floor muscles five times in five sets, with a 10-s rest between each set, and electromyography data on muscle strength, fatigue, and coordination of type II muscle fibers in the pelvic region were gathered.

Pelvic floor muscle contractions were held for 6 s, repeated in five sets with a 10-s interval between each set, and electromyographic data on muscle strength, fatigue, and pelvic floor coordination of type I muscle fibers were collected.

### Specific steps for POP-Q staging

The staging was performed with the patient straining so that the maximum descent was attained.

In a quiet environment, after voluntarily emptying their bladder, the participant took the lithotomy position, disinfected the vulva and urethral meatus, and performed the Valsalva maneuver (Frawley et al., 2021) for 6 s (forceful exhalation against a closed mouth, glottis, and nose). With the hymen edge as reference (0 points), the examiner observed six indicator points on the anterior wall, posterior wall, and apex of the vagina (two points Aa, Ba on the anterior wall, two points Ap, Bp on the posterior wall, and two points C, and D on the apex). The distance of these six points relative to the hymen edge was represented numerically, with the inner side of the hymen edge recorded as negative and the outer side as positive.

### Specific steps for stress testing and Marshall–Bonney testing

The doctor asked the patient in the bladder filling status to cough while observing the urethral opening for any involuntary urine leakage. If there was leakage during coughing in the lithotomy position, further Marshall–Bonney testing was conducted. The doctor placed the middle and index fingers on both sides of the urethra in the anterior vaginal wall, with the fingertips at the junction of the bladder and urethra. Then, the doctor lifted the bladder neck forward and induced stress testing. If there was no leakage at this point, the test was positive.

3. The questionnaires were collected, and the data were screened and examined. The incidence of POP and UI in the early postpartum period and the changes in pelvic floor EP were investigated. The demographic characteristics and obstetric risk factors were also examined.

**FIGURE 1:**
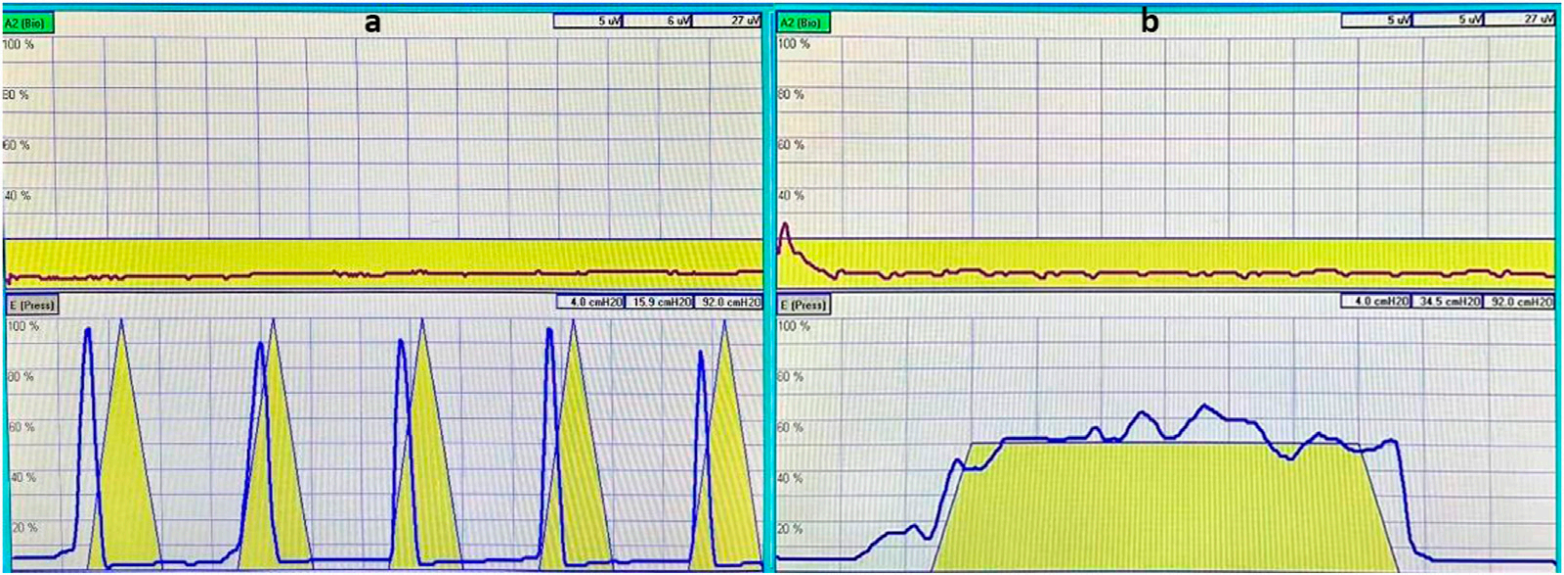
The yellow triangle pattern in the image is a template for the instrument’s muscle contraction to prompt the patient to contract and relax, as indicated in the pattern. The red curve in the upper image shows the patient’s synchronously measured strength of abdominal muscle contraction, which is used to detect whether there is compensation from abdominal muscles during pelvic floor muscle contractions. The blue curve in the aforementioned image shows the strength of contraction of type II muscle fibers in the patient’s pelvic floor muscles **(A)**; the yellow trapezoidal pattern is the instrument’s muscle contraction template, which is designed to prompt the patients to sustain the contraction, as indicated in the diagram before relaxing. The red curve in the upper figure is the patient’s synchronous measurement of the abdominal muscle contraction strength curve, used to detect whether there is a compensatory contraction of the abdominal muscles during the pelvic floor muscle contraction process. The blue curve in the aforementioned figure is the patient’s pelvic floor muscle type I fiber contraction strength curve **(B)**.

Observation indicator:

1. Measurement of muscle strength (Xing et al., 2015; Yang et al., 2019): The puerperae contracted their pelvic floor muscles to pressurize the vaginal balloon probe, the difference in pressure was measured, and the muscle strength of type I and II pelvic floor muscles was evaluated. The strength of type II pelvic floor muscles was evaluated based on the number of consecutive contractions that reached at least 60% of the maximum contraction intensity determined by the contraction curve. The number of consecutive contractions corresponding to grade 0–5 muscle strength was 0, 1, 2, 3, 4, and 5, respectively; the muscle strength of ≥ grade 3 was normal, and the grade 0–2 muscle strength was decreased (Figure 1A). The strength of type I pelvic floor muscles was evaluated based on the duration of sustained contractions that reached at least 40% of the maximum contraction intensity determined by the contraction curve. Contraction duration corresponding to grade 0–5 muscle strength was 0, 1, 2, 3, 4, and ≥5 s, respectively (Figure 1B).
2. Muscle fatigue degree (Xing et al., 2015; Yang et al., 2019): Type I pelvic floor muscle fatigue degree was the percentage of the descending distance between the highest peak point within the first 1 s and the highest point at the endpoint of 6 s on the contraction curve, and the type II pelvic floor muscle fatigue degree was the percentage of the descending distance I between the first peak point and the fifth peak point on the contraction curve; 0 was normal, and a negative value was abnormal.
3. Abnormal results of pelvic floor EP examination: defined as type I or II pelvic floor muscle fiber abnormalities involving muscle strength and fatigue degree;
4. Vaginal dynamic pressure: The vaginal dynamic pressure was measured using a balloon probe, with a normal range being 80–150 cm H_2_O.
5. POP (Haylen et al., 2016; Akın et al., 2018): Downward displacements of one or more areas, such as the anterior vaginal wall, posterior vaginal wall, and uterus (cervix) or vaginal apex, were defined anatomically as anterior POP, posterior POP, and apical POP, respectively. The degree of prolapse was quantified and classified into five stages according to POP-Q (Persu et al., 2011) (Table 1).
6. Urinary incontinence (UI) (Abrams et al., 2003): UI was mainly stress urinary incontinence, and the diagnosis relied on the patient’s urinary incontinence history, urinary incontinence questionnaire (ICI-Q-SF), and gynecological examination, as well as stress testing and Marshall–Bonney testing. The definition refers to the International Continence Society’s standard (Gajewski et al., 2018): Urine involuntarily leaks when abdominal pressure increases.

**TABLE 1:** POP-Q classification and staging criteria.

Stage	Criteria
0	There is no prolapse, and Aa, Ba, Ap, and Bp are measured at −3 cm, and point C is between −tvl and −(tvl-2) cm
I	The furthest point of prolapse is within the vagina, located within a range of −3 to −1 cm from the hymen
II	The furthest point of prolapse is within a range of 1 to −1 cm from the edge of the hymen
III	The furthest point of prolapse is outside of the hymen, with a distance ranging from +1 to (tvl-2) cm from the edge of the hymen
IV	The lower genital tract is completely or almost completely protruding outside the hymen, and the furthest point of prolapse is ≥(tvl-2) cm

Note: tvl: The total length from the top of the vagina to the edge of the hymen when c and d are in their normal positions (range 10 cm–12 cm).

### Statistical methods

SPSS 19.0 software was used for statistical analysis. The measurement data were presented as mean ± standard deviation, and the counting data were presented as percentage or incidence rate. Multifactor analyses with POP and UI as dependent variables were conducted using binary logistic expression analysis, and single-factor analyses with age, BMI, gravidity, parity, frequency of pregnancy loss, and fetal birth weight as independent variables were conducted using the Mann–Whitney *U* test. Chi-squared analysis was used to conduct a single-factor analysis with delivery mode as the independent variable. Chi-squared analysis was used to compare the incidence of decreased muscle strength and abnormal fatigue degree of pelvic floor muscles among women with different types of POP and UI. The difference in vaginal dynamic pressure among women with different types of POP and UI was assessed using the Kruskal–Wallis H test. A pairwise comparison was performed using the Mann–Whitney *U* test and Bonferroni’s correction. *p* < 0.05 indicated a statistically significant difference.

## Results

A total of 1,539 puerperae were enrolled in this study. Among them, 65 had incomplete data, of whom the data of 42 puerperae were supplemented by telephone follow-up, and 23 puerperae with incomplete data (13 had no data on fatigue degree, 3 had no data on gravidity and parity, 5 had no data on UI, and 2 had non-staged POP) were excluded. The data on the remaining 1,516 puerperae were included in the analysis. Table 2 shows the basic demographic characteristics and the current obstetric data on the included women; 85.3% of them had a delivery age between 26 and 35 years, and 41.8% of them underwent cesarean section.

**TABLE 2:** General characteristics of women at 6–8 weeks after delivery and obstetrical data during this pregnancy; N = 1,516.

Characteristic variable	Percentage and incidence rate (%,n/N)	Range	Mean ± SD
Delivery age (years)			
≤25	17.2 (261/1,516)	19–49	28.8 ± 3.9
26 ≤ 30	56.8 (861/1,516)		
31 ≤ 35	18.5 (280/1,516)		
36 ≤ 40	6.8 (103/1,516)		
>40	0.7 (11/1,516)		
BMI (kg/m^2^)			
≤18.4	2.4 (37/1,516)	16.0–37.5	23.5 ± 3.1
18.5 ≤ 24.9	69.3 (1,051/1,516)		
25 ≤ 29.9	24.5 (372/1,516)		
>30	3.7 (56/1,516)		
Gravidity (time)			
1	56.7 (860/1,516)	1–8	1.7 ± 1.0
2	24.2 (367/1,516)		
3	12.5 (190/1,516)		
≥4	6.5 (99/1,516)		
Parity (time)			
1	73.5 (1,115/1,516)	1–4	1.3 ± 0.5
2	25.7 (390/1,516)		
3	0.7 (10/1,516)		
≥4	0.0 (1/1,516)		
Delivery mode			
Vaginal delivery	58.2 (883/1,516)	—	—
Cesarean section(633/1,516)	41.8		
Fetal birth weight (kg)			
<2.5	4.0 (60/1,516)	1.2–5.1	3.3 ± 0.5
2.5–4.0	88.4 (1,340/1,516)		
≥4.0	7.7 (116/1,516)		

Note: SD, standard deviation; BMI, body mass index. Data are presented as a percentage of the total number (%, n/N), range, and mean ± standard deviation (mean ± SD).

Tables 3, 4 show the incidence of PFD and results of pelvic floor muscle EP examination in postpartum women at 6–8 weeks. As shown in Table 3, among the 1,516 puerperae, 1,275 had clinical symptoms of PFD, and the incidence rate was 84.1%. The incidence rates of various types of POP and UI ranked from high to low, respectively: anterior POP (78.8%), UI during this pregnancy (18.8%), posterior POP (13.5%), UI during this postpartum period (11.8%), and apical POP (7.8%). Of the 1,516 puerperae , 1,443 had abnormal pelvic floor muscle EP, and the incidence was 95.2%. As shown in Table 4, the incidence of decreased muscle strength and abnormal fatigue degree in type I pelvic floor muscles were significantly higher than in type II pelvic floor muscles, and the differences were statistically significant (*p* < 0.05).

**TABLE 3:** Data on incidence rates of POP and UI in 1,516 women at 6–8 weeks after delivery.

Classification of PFDs	Cases (n)	Percentage (%, n/1,275)	Incidence rate (%,n/1,516)
POP	1,241	97.3	81.9
Anterior (anterior vaginal wall)	1,194	93.6	78.8
Stage I	1,123	88.1	74.1
Stage II	71	5.6	4.7
Posterior (posterior vaginal wall)	204	16.0	13.5
Stage I	197	15.5	13.0
Stage II	7	0.5	0.5
Apical (downward displacement of the uterine neck)	119	9.3	7.8
UI	387	30.4	25.5
Only during this pregnancy	208	16.3	13.7
Only after this pregnancy	101	7.9	6.7
During and after this pregnancy	78	6.1	5.1
Total	1,275	100	84.1

Note: POP, pelvic organ prolapse; UI, urinary incontinence; PFD, pelvic floor dysfunction.

**TABLE 4:** EP data on pelvic floor muscles of women at 6–8 weeks after delivery; N = 1,516.

Pelvic floor muscle	Muscle strength^a^						Fatigue degree^a^	
Type I	0	I	II	III	IV	V	0	<0
	15	875	440	159	27	0	212 (14.0%)	1,304 (86.0%)^②^
	1,330 (87.7%)^①^			186 (12.3%)				
Type II	0	I	II	III	IV	V	0	<0
	3	282	336	661	222	12	1,364 (90.0%)	152 (10.0%)^④^
	621 (41.0%)^③^			895 (59.0%)				
Vaginal dynamic pressure^b^	73.9 ± 14.3 (cmH_2_O)							

^a^ The counting data are presented as the incidence rate (%, n/N), and the comparison was performed using chi-squared analysis.

^b^ The measurement data are presented as mean ± SD.

*p* < 0.05: ①vs.③; ①vs.④; ②vs.③; ②vs.④; ③vs.④.

We conducted single-factor and multifactor analyses on various types of POP and UI among the six groups of postpartum women at 6–8 weeks. As shown in Tables 5A–F, anterior POP, posterior POP, anterior and posterior stage II POP, and postpartum UI were more likely to appear in puerperae who had vaginal deliveries. In the single-factor analysis of apical POP, there was a statistical difference in gravidity but no statistical difference in parity. Therefore, in the multifactor regression equation, the frequency of pregnancy loss instead of parity was used as an independent variable in the analysis. The results of this study revealed that pregnancy loss was a risk factor for apical POP (because the incidence of premature birth was very low, the pregnancy loss occurred mainly due to abortion; no detailed classification was performed here).

**TABLE 5:** Single-factor and multifactor analyses with PFDs as dependent variables and general conditions and obstetrical characteristics of parturient as independent variables **(A–F)**: **POP, pelvic organ prolapse; UI, urinary incontinence; PFD, pelvic floor dysfunction; OR, odds ratio; CI, confidence interval.**

(A) Anterior POP					
PFD/factor	Single-factor analysis			Multifactor analysis	
	Yes	No	*p*-value (Z/χ^2^)	OR (95% CI)	*p*-value
Anterior POP	1,194	322			
Age	28.7 ± 3.8	29.3 ± 4.2	0.034 (2.120)	0.999 (0.961–1.039)	0.973
BMI	23.4 ± 3.0	23.7 ± 3.4	0.281 (1.078)	1.018 (0.976–1.062)	0.408
Gravidity	1.7 ± 1.0	1.8 ± 1.1	0.031 (2.156)	0.950 (0.804–1.122)	0.547
Parity	1.3 ± 0.5	1.3 ± 0.4	0.499 (0.676)	1.546 (1.052–2.271)	0.027
Vaginal delivery	91.7% (810/883)	8.3% (73/883)	0.000 (212.747)	7.850 (5.804–10.617)	0.000
Fetal birth weight	3.3 ± 0.5	3.3 ± 0.5	0.461 (0.737)	1.092 (0.838–1.425)	0.514

(B) Posterior POP					
PFD/factor	Single-factor analysis			Multifactor analysis	
	Yes	No	*p*-value (Z/χ^2^)	OR (95% CI)	*p*-value
Posterior POP	204	1,312			
Age	29.3 ± 3.8	28.8 ± 3.9	0.033 (2.128)	1.056 (1.007–1.108)	0.026
BMI	23.6 ± 2.9	23.5 ± 3.2	0.546 (0.604)	1.039 (0.987–1.094)	0.143
Gravidity	1.8 ± 1.1	1.7 ± 1.0	0.351 (0.932)	1.013 (0.826–1.243)	0.899
Parity	1.3 ± 0.5	1.3 ± 0.5	0.076 (1.772)	1.397 (0.889–2.198)	0.147
Vaginal delivery	19.5% (172/883)	80.5 (711/883)	0.000 (65.865)	5.990 (3.953–9.077)	0.000
Fetal birth weight	3.4 ± 0.5	3.3 ± 0.5	0.076 (1.776)	1.465 (1.041–2.062)	0.028

(C) Apical POP					
PFD/factor	Single-factor analysis			Multifactor analysis	
	Yes	No	*p*-value (Z/χ^2^)	OR (95% CI)	*p*-value
Apical POP	119	1,397			
Age	28.9 ± 4.7	28.8 ± 3.8	0.588 (0.542)	0.989 (0.933–1.048)	0.702
BMI	23.5 ± 3.1	23.5 ± 3.1	0.752 (0.316)	1.003 (0.943–1.067)	0.902
Gravidity	1.9 ± 1.1	1.7 ± 1.0	0.013 (2.494)	0.771 (0.467–1.271)	0.308
Parity	1.2 ± 0.4	1.3 ± 0.5	0.645 (0.461)	—	—
Frequency of pregnancy loss	0.7 ± 1.0	0.4 ± 0.8	0.000 (3.538)	1.853 (1.060–3.237)	0.030
Vaginal delivery	7.8% (69/883)	92.2% (814/883)	0.952 (0.004)	1.023 (0.686–1.527)	0.909
Fetal birth weight	3.3 ± 0.5	3.3 ± 0.5	0.314 (1.007)	0.803 (0.546–1.181)	0.265

(D) Stage II POP (anterior and posterior)					
PFD/factor	Single-factor analysis			Multifactor analysis	
	Yes	No	*p*-value (Z/χ^2^)	OR (95% CI)	*p*-value
Stage II POP (anterior and posterior)	74	1,442			
Age	29.5 ± 3.6	28.8 ± 3.9	0.047 (1.989)	1.020 (0.946–1.100)	0.611
BMI	23.4 ± 3.0	23.5 ± 3.1	0.980 (0.025)	1.008 (0.928–1.095)	0.847
Gravidity	1.9 ± 1.0	1.7 ± 1.0	0.015 (2.433)	0.824 (0.578–1.174)	0.284
Parity	1.5 ± 0.5	1.3 ± 0.5	0.000 (4.486)	4.162 (2.125–8.152)	0.000
Vaginal delivery	7.4% (65/883)	92.6% (818/883)	0.000 (28.013)	7.636 (3.662–15.919)	0.000
Fetal birth weight	3.4 ± 0.4	3.3 ± 0.5	0.856 (0.181)	1.113 (0.656–1.886)	0.692

(E) UI during this pregnancy					
PFD/factor	Single-factor analysis			Multifactor analysis	
	Yes	No	*p*-value (Z/χ^2^)	OR (95% CI)	*p*-value
UI during this pregnancy	286	1,230			
Age	29.3 ± 3.9	28.7 ± 3.9	0.006 (2.738)	1.038 (0.999–1.079)	0.055
BMI	23.6 ± 3.2	23.4 ± 3.1	0.378 (0.881)	1.013 (0.972–1.057)	0.531
Gravidity	1.8 ± 1.1	1.7 ± 1.0	0.148 (1.448)	1.051 (0.892–1.239)	0.550
Parity	1.3 ± 0.5	1.3 ± 0.5	0.269 (1.104)	0.891 (0.610–1.301)	0.550
Fetal birth weight	3.3 ± 0.5	3.3 ± 0.5	0.342 (0.949)	1.049 (0.801–1.374)	0.727

(F) UI after this pregnancy					
PFD/factor	Single-factor analysis			Multifactor analysis	
	Yes	No	*p*-value (Z/χ^2^)	OR (95% CI)	*p*-value
UI after this pregnancy	179	1,337			
Age	29.3 ± 3.8	28.8 ± 3.9	0.028 (2.199)	1.066 (1.014–1.120)	0.013
BMI	24.1 ± 3.3	23.4 ± 3.1	0.003 (2.982)	1.117 (1.060–1.177)	0.000
Gravidity	1.8 ± 1.1	1.7 ± 1.0	0.740 (0.331)	1.064 (0.863–1.312)	0.561
Parity	1.3 ± 0.5	1.3 ± 0.5	0.267 (1.111)	1.030 (0.635–1.672)	0.905
Vaginal delivery	17.0% (150/883)	83.0% (733/883)	0.000 (54.495)	6.046 (3.894–9.387)	0.000
Fetal birth weight	3.4 ± 0.4	3.3 ± 0.5	0.337 (0.960)	1.236 (0.870–1.758)	0.237

Table 6 shows the comparison of pelvic floor muscle EP among women with different types of POP and UI. Among women with various types of POP and UI, the incidence of decreased muscle strength in type I and II pelvic floor muscles in women with anterior and posterior stage II POP was higher than those in the other groups; the differences were statistically significant, *p* < 0.05, and there was no significant difference among other groups, all with *p* > 0.05. The incidence of abnormal fatigue degree in type I pelvic floor muscles in women with postpartum UI was significantly higher than in women with apical POP, posterior POP, and UI during this pregnancy, and it was higher in women with anterior POP than in women with apical POP, and the differences were statistically significant (*p* < 0.05); there was no significant difference between the other groups, *p* > 0.05. The incidence of abnormal fatigue degree in type II pelvic floor muscles was higher in women with anterior and posterior stage II POP than in the other groups; it was higher in women with apical POP than in women with UI during this pregnancy, anterior POP, and postpartum UI, and higher in women with posterior POP than in women with UI during this pregnancy and anterior POP; the differences were statistically significant (*p* < 0.05); there was no statistical significance among other groups, *p* > 0.05.

**TABLE 6:** EP data on different types of PFD in women at 6–8 weeks after delivery.

Classification of PFDs	POP				UI	
	Anterior	Posterior	Apical	Stage II (anterior and posterior)	During this pregnancy	After this pregnancy
Total number of cases	1,194	204	119	74	286	179
Type I muscle^a^						
0–2	89.1% (1,064/1,194)	88.8% (181/204)	84.0% (100/119)	98.6% (73/74)	88.1% (252/286)	87.7% (157/179)
3–5	10.9% (130/1,194)	11.3% (23/204)	16.0% (19/119)	1.4% (1/74)	11.9% (34/286)	12.3% (22/179)
Fatigue degree <0	86.8% (1,036/1,194)	84.3% (172/204)	78.2% (93/119)	86.5% (64/74)	85.0% (243/286)	91.6% (164/179)
Type II muscle^a^						
0–2	43.1% (515/1,194)	49.5% (101/204)	46.2% (55/119)	73.0% (54/74)	44.4% (127/286)	48.6% (87/179)
3–5	56.9% (679/1,194)	50.5% (103/204)	53.8% (64/119)	27.0% (20/74)	55.6% (159/286)	51.4% (92/179)
Fatigue degree<0	11.4% (136/1,194)	18.1% (37/204)	21.8% (26/119)	37.8% (28/74)	10.5% (30/286)	12.3% (22/179)
Vaginal dynamic pressure^b^ (cmH_2_O)	73.1 ± 13.5	72.9 ± 14.1	74.2 ± 13.7	67.9 ± 11.4	73.2 ± 13.8	73.3 ± 15.6

Note: PFD, pelvic floor dysfunction; POP, pelvic organ prolapse; UI, urinary incontinence.

^a^ The counting data are presented as the incidence rate (%, n/N), and the comparison was performed using chi-squared analysis.

^b^ The measurement data are presented as (mean ± SD), and comparison among multiple groups was performed using the Kruskal–Wallis H test.

A pairwise comparison was performed using the Mann–Whitney *U* test and Bonferroni’s correction.

There were statistically significant differences in vaginal dynamic pressure among women with different types of POP and UI (χ^2^ = 13.120, *p* = 0.022), *p* < 0.05; the pairwise comparison showed that the vaginal dynamic pressure of women with anterior and posterior stage II POP was lower than those of women with anterior POP (*p* = 0.001), apical POP (*p* = 0.001), and UI during this pregnancy (*p* = 0.002), and the differences were statistically significant, *p* < 0.003.

## Discussion

PFD includes lower urinary tract dysfunction, organ prolapse, sexual dysfunction, abnormal defecation, and chronic pelvic pain, of which stress UI, vaginal anterior and posterior prolapse, and uterine prolapse are most commonly seen (Zuchelo et al., 2018; Pandeva et al., 2019). Approximately 24% of adult women have at least one PFD symptom. With an increase in age, the incidence of PFD in women aged 65–70 years and over 80 years reaches 40% and 50%, respectively (Nygaard et al., 2008; Hoen et al., 2015). A study showed that the risk of occurrence of grade 1–3 POP in American women aged 50–79 was 41.1% (Doumouchtsis and Chrysanthopoulou, 2013). The prevalence of POP in the general female population is about 3.4%–56.4% (Walker and Gunasekera, 2011), the lifetime risk of POP is 11%–19% (Smith et al., 2010), and in American women aged 40–50 years, the prevalence rate of UI is 17.2%, and in American women aged over 80 years, it is 31.7% (Nygaard et al., 2008). As for the incidence of postpartum PFD, a survey of 264 pregnant women in Beijing in 2014 showed that the incidence of UI was 18%–27% in women after vaginal delivery and 13%–20% in women after cesarean section (Li et al., 2014). A prospective study of 284 pregnant women conducted by Reimers et al. showed that the incidence of stage II POP was 8.8% and the incidence of vaginal wall prolapse was 16%–23% in women at 6 weeks after delivery (Reimers et al., 2018). In this study, the incidence of UI was 25.5%, the incidence of stage II anterior and posterior vaginal wall prolapse was 4.9%, and the incidence of apical POP was 7.8% in postpartum women at 6–8 weeks, which were consistent with the results in the aforementioned studies. The total incidence of POP was as high as 81.9%, which was attributable to the staging method, reversibility, and dynamic characteristics of prolapse.

The core of the pelvic floor muscle EP examination is the electrical diagnosis of the main nerve and muscle functions of the pelvic floor. After pregnancy and delivery, if inducements and risk factors continue to exist, muscle cells are damaged when the threshold of pelvic floor function is exceeded. First, the fatigue degree will be abnormal, followed by changes in the tissue biology and pelvic abdominal dynamics and different degrees of decreased muscle strength. A study by Allen et al. using EP indicated reinnervation of the pelvic floor muscles in 80% of women after vaginal delivery (South et al., 2009). In 2015, Xing et al. (2015) found that the incidence of abnormal pelvic floor muscle EP was 92.3% in postpartum women at 6–8 weeks, who were not provided with any analgesia during labor and the incidence rates of declined muscle strength and abnormal fatigue degree in type I and II pelvic floor muscles were 85.9%, 51.4%, 75.4%, and 12.7% respectively. Another study of pelvic floor EP in 5,143 Chinese puerperae (Yang et al., 2019) showed that approximately half of the women had abnormal EP indicators in the early postpartum period. In this study, the incidence of abnormal pelvic floor muscle EP in 1,516 women in the early postpartum period was 95.2%. Although the results are not completely consistent, which may be attributable to the difference in the time and method of examination, they indicate that pregnancy and delivery could lead to potential pelvic floor injuries. Changes in pelvic floor electrophysiological indices appear before the onset of pelvic floor symptoms and are significantly correlated with PFD, providing a more accurate and objective reflection of the condition of the pelvic floor muscles. They are sensitive indicators of early pelvic floor tissue damage.

Pelvic floor muscles can be divided into two types according to their characteristics: type I pelvic floor muscle fibers mainly exist in the levator ani muscles; their contraction can last for a long time, and they are not easily subjected to fatigue. Their main function is maintaining the pelvic and abdominal organs in the normal position representing the pelvic support system. Type II pelvic floor muscle fibers mainly exist in the superficial perineal muscles; their contraction lasts for a short time, and they are easily fatigued. Their main function is to aid in urine control representing the pelvic motor system (Aljuraifani et al., 2019). In this study, the abnormal rates of muscle strength and fatigue degree of type I pelvic floor muscles were significantly higher than those of type II pelvic floor muscles, and it was considered that the support system was more affected. In fact, the results suggested that the incidence of POP was much higher than that of stress urinary incontinence (SUI). At the same time, it was also indicated that pregnancy and delivery might cause significant damage to the levator ani muscle, which has been confirmed (Lee et al., 2005; Ian and Maria, 2018; Blomquist et al., 2019). It is worth noting that, unlike POP, UI often occurs sporadically during the perinatal period, which indicates that some of the type II pelvic floor muscle abnormalities in the early postpartum period are physiological or reversible, and it is necessary for puerperae to undergo re-examination at 3 months post-delivery. Perhaps the postpartum period could be a favorable period and could be taken as a critical period for early intervention into UI age progression (Zuchelo et al., 2018). A follow-up study is required to further analyze the EP data on women at 3–6 months or even longer postpartum.

Many risk factors for development and symptom progression have been identified, including pregnancy and delivery (Groutz et al., 2004; Sievert et al., 2012), age (Maclennan et al., 2000; Mannella et al., 2013), obesity (Moreno-Vecino et al., 2015; MacArthur et al., 2016; Brucker et al., 2017; Barbosa et al., 2018b; Islam et al., 2018; Marcelissen et al., 2019), and delivery mode (Ashton-Miller and Delancey, 2009; Slieker-ten Hove et al., 2009; Buurman and Lagro-Janssen, 2013). Many of the risk factors are shared for POP and UI. First, age is a recognized factor affecting the anatomy and function of the pelvic floor and lower urinary tract. The gradual loss of the elasticity of the pelvic floor connective tissue should be the cause of the gradual increase of POP and UI in the process of aging; however, the exact mechanism and pathological process are not completely clear (Doumouchtsis and Chrysanthopoulou, 2013). In this study, the multifactor analysis revealed that posterior POP and postpartum UI were correlated with age, while other types of PFD were not detected. Meanwhile, we did not find any common changes in pelvic floor muscle EP between the aforementioned two types of PFD. We considered whether the muscle fibers and nerves in the urethra or the posterior vaginal wall were more susceptible by age in comparison with other types of PFD. Of course, the majority of the puerperae in this study were aged between 25 and 35 years, and it is possible that this bias could reduce the influence of age on all types of PFD. Many studies (Moreno-Vecino et al., 2015; MacArthur et al., 2016; Brucker et al., 2017; Barbosa et al., 2018b; Islam et al., 2018; Marcelissen et al., 2019; Tennfjord et al., 2020) have confirmed the repercussions of obesity on UI and even confirmed the protective effect of conservative weight loss on UI.

In this study, we found that BMI was a risk factor for postpartum UI. The result of parity on PFD often interacts with vaginal delivery. Pelvic floor function and anatomical damage (Ashton-Miller and Delancey, 2009; Slieker-ten Hove et al., 2009) are more likely caused by vaginal delivery and multiparity. However, the long-term protective effect of cesarean section is still controversial (Friedman et al., 2012; Li et al., 2014; Qian et al., 2016). At the same time, considering the possible complications during and after cesarean section (Li et al., 2014), it is not recommended to perform elective cesarean section only to avoid the occurrence of PFD. In this study, the effect of frequency of pregnancy loss instead of parity on apical POP was investigated. As the incidence of premature birth was very low, pregnancy loss occurred mainly due to abortion, and no detailed classification was carried out here. It was found that pregnancy loss was the only risk factor for apical POP among the abovementioned multiple factors, and neither gravidity nor delivery mode were the only risk factors for apical POP. The causes were investigated as follows: apical POP was more affected by the pelvic ligaments and nerves than the pelvic floor and was more affected by pregnancy than the delivery process. In terms of fetal birth weight, no correlation was observed between fetal birth weight and UI during pregnancy, but a correlation was observed between fetal birth weight and posterior POP. It can be further studied whether this is related to the fetal connection and the traction and compression effect of the fetal head on the vaginal wall during labor and whether there is a correlation between the time duration between the fetal head descending into the pelvis and the starting time of the first stage of labor with the times of the three stages of labor.

## Conclusion

PFD is a complex process with multiple and multifactorial etiology. The interaction of various factors such as age, gravidity, parity, delivery mode, and fetal birth weight leads to postpartum PFD. Pelvic floor muscle EP is a sensitive indicator of early pelvic floor injury. In most cases, the changes in muscle strength and fatigue degree coexist in different types of postpartum PFD, and each has its respective characteristics that need to be evaluated using additional examination data.

## Data Availability

The raw data supporting the conclusion of this article will be made available by the authors, without undue reservation.

## Abbreviations

BMI, body mass index; CI, confidence interval; EP, electrophysiology; MRI, magnetic resonance imaging; OR, odds ratio; PFD, pelvic floor dysfunction; POP, pelvic organ prolapse; POP-Q, pelvic organ prolapse quantitation; SD, standard deviation; SUI, stress urinary incontinence; UI, urinary incontinence.
